# Healthcare Expatriate Adjustment in Qatar: Analyzing Challenges and Opportunities

**DOI:** 10.7759/cureus.54720

**Published:** 2024-02-22

**Authors:** Ayesha Bashir

**Affiliations:** 1 International Business Management, Business School, University of Edinburgh, Edinburgh, GBR

**Keywords:** bureaucratic challenges, language barriers, cultural integration, healthcare professionals, expatriate adjustment

## Abstract

Background: The healthcare sector in the Middle East, particularly in Qatar, relies heavily on expatriate professionals. The successful adaptation of these expatriates to the local environment is crucial as it significantly affects their job satisfaction and the quality of care they deliver to patients. This study aimed to identify and analyze the key factors influencing the adjustment of healthcare expatriates in Qatar to their professional roles and the cultural context of the region.

Methodology: This study involved the use of a cross-sectional survey approach targeting healthcare professionals in Qatar, with a targeted sample size of 385 participants selected to ensure a comprehensive representation of the expatriate workforce. The survey was disseminated through expatriate-specific WhatsApp groups from October 1, 2022, to December 31, 2022. It was structured to examine a variety of factors influencing expatriate adjustment, including age, gender, proficiency in the Arabic language, social support networks, job type, and length of stay in Qatar. The primary focus of this study is a thematic analysis of the respondents' free-text comments. These comments offer valuable insights into their experiences, both positive and negative, in adapting to life and work in Qatar.

Results: This study achieved a response rate of 51.69% (199 out of 385), with participants representing 10 nationalities, being predominantly aged between 35 and 44 years, and having spent four to seven years in Qatar. The majority of respondents were nurses and doctors, accounting for 83.9% of the sample. The following five main themes emerged from the analysis of 218 free-text comments: personal and family concerns, work-related matters, government and system-related challenges, social and cultural challenges, and Arabic language challenges. Although participants valued supportive employer practices in family relocation and professional environments, they encountered language barriers, bureaucratic complexities, and cultural adaptation challenges.

Conclusion: The results of this study shed light on the multifaceted nature of expatriate adjustment in the healthcare sector of Qatar, highlighting the supportive elements and the obstacles encountered. They emphasize the need for comprehensive support mechanisms, including language training, streamlined bureaucratic processes, and cultural orientation programs, to facilitate successful and fulfilling expatriate experiences in the Middle Eastern healthcare context.

## Introduction

Expatriates are professionals from one country who work in another country. Expatriate healthcare workers are pivotal in the healthcare sectors of their host countries [1]. Their roles include filling skill gaps with specialized knowledge that may be scarce locally, thereby elevating the quality of healthcare services [2]. Additionally, they facilitate knowledge transfer, enhancing the capabilities of local healthcare workers and fostering educational growth within the community [3]. These professionals also bring diverse cultural perspectives, which are beneficial in treating a diverse patient demographic and shaping inclusive healthcare policies [4]. Expatriates address workforce shortages, particularly in regions with aging populations and rural areas, ensuring the population’s healthcare needs are met [5]. Moreover, their involvement in research and the introduction of innovative practices and technologies drives advancements in the healthcare sector [6]. Overall, expatriates are instrumental in enhancing the host country’s healthcare capabilities, ensuring access to high-quality care for patients, and contributing to the sector’s continuous improvement and evolution [7].

Expatriates constitute a significant portion of Qatar’s total population, playing a vital role in the country’s healthcare system, which boasts prestigious Joint Commission International (JCI) and Accreditation Council for Graduate Medical Education-International (ACGME-I) accreditations [8]. The presence of expatriates in Qatar’s healthcare system is crucial because they fill essential roles, provide specialized services, and bring a global perspective to medical and patient care practices [9]. The effectiveness of these expatriate professionals significantly hinges on their ability to adjust to the local culture and healthcare environment [5]. This adjustment is essential for seamless integration into the healthcare system, ensuring they can function optimally within Qatar’s unique healthcare landscape, thereby contributing to the nation’s healthcare sector [10].

Research into expatriate adjustment has identified a significant gap in understanding the determinants of adjustment within the healthcare sector in the Middle Eastern context. Despite a wealth of studies on expatriates across various industries, investigations specifically addressing healthcare professionals in this region are notably limited [11,12]. This dearth of in-depth research is particularly important given the distinctive challenges and experiences that healthcare expatriates encounter in the Middle East, which are likely to vary substantially from those in other sectors or geographical areas [13,14]. Therefore, this gap highlights an imperative need for targeted research that explores the experiences and unique challenges faced by healthcare expatriates in the Middle Eastern setting, thereby explaining the complexities of their integration into this specific cultural and professional environment [15,16].

## Materials and methods

This study, part of a master's thesis approved by the Institutional Review Board of the University of Edinburgh, Scotland (reference #B210352), employed a cross-sectional thematic analysis to understand the adjustment experiences of expatriate healthcare professionals in Qatar.

Survey design and distribution

A detailed survey was designed and distributed to a purposive sample of expatriate healthcare workers in Qatar, including roles such as doctors, nurses, administrators, and clerical staff across public and private sectors. This survey was conducted in the duration from October 1, 2022, to December 31, 2022, which aimed to capture diverse perspectives within Qatar’s healthcare sector, with weekly reminders sent to expatriate-specific WhatsApp groups to encourage participation.

Demographics of participants

The survey collected demographic and social attributes of the participants. This included information on gender, nationality, age groups, duration of stay in Qatar, job titles, and Arabic language proficiency. The diversity of participants provided a rich, varied understanding of expatriate experiences in the healthcare sector.

Sample size and response rate

Initially targeting 385 participants for a 95% confidence level with a ±5% margin of error, the study ultimately secured 199 complete responses due to practical constraints. This sample size was deemed sufficient to provide insightful qualitative analysis.

Thematic analysis of responses

Our primary focus was on the thematic analysis of free-text responses, offering qualitative insights into the expatriates' experiences. We meticulously reviewed, coded, and categorized these responses into major themes. Furthermore, we derived recommendations for healthcare-employing organizations in Qatar based on these themes, aiming to improve the experiences of expatriates. This thematic exploration provided a detailed understanding of both the positive and negative aspects of expatriate life in Qatar's healthcare sector

Questionnaire development

The questionnaire for this study, detailed in the table in the appendix, was rigorously developed following an extensive review of the literature on expatriate adjustment. It includes sections to gather demographic data, job-related factors, language proficiency in the host country, and the extent of social support available to the expatriates. A five-point Likert scale was employed to gauge levels of adjustment and the quality of social support.

The design of the questionnaire was informed by the need to understand the multifaceted nature of expatriate life in Qatar. Questions were tailored to capture the details of their experiences, ranging from basic demographic information to more subjective assessments of their adjustment and well-being in various aspects of life and work.

Questionnaire features

The questionnaire in the table in the appendix begins with basic demographic questions, including gender, age, nationality, occupational level, and language proficiency. This is followed by a series of questions on experiences within the company, such as the availability of companionship for necessary activities, access to information for orientation, and assistance with local regulations (items 1-12). Respondents rate these on a five-point scale from "no one" to "many would do this."

Further, the questionnaire probes into the expatriates' adjustment in Qatar, covering living conditions, housing quality, access to amenities, and social interactions with locals. These aspects are also rated on a five-point scale from "extremely adjusted" to "extremely unadjusted." The questionnaire's design and structure are aimed at eliciting comprehensive and detailed responses, thereby enabling a detailed analysis of expatriate adjustment experiences.

Pilot testing and revision

Before finalizing the questionnaire, it underwent pilot testing with a targeted group of five expatriates. Feedback from this pilot phase was used to make necessary adjustments. The revised questionnaire was then reviewed by a research supervisor with expertise in expatriate research and cross-cultural psychology, ensuring its relevance and effectiveness in capturing the intended data.

## Results

Demographic overview

The present study analyzed 199 expatriate healthcare workers in Qatar, revealing a nearly balanced gender distribution with 45.70% male and 54.30% female participants. The age group most represented was 35-44 years (38.70%), followed by 45-54 years (34.20%). The sample showcased Qatar's healthcare sector's multicultural nature, with significant Filipino (23.10%), Indian (17.10%), and Egyptian (5.50%) representation. The duration of stay in Qatar varied, indicating diverse lengths of professional experience. A large segment had been living in Qatar for four to seven years (39.20%) and one to three years (35.70%). The majority of participants were in professional roles like nurses and doctors (83.90%), with varying Arabic language proficiency levels, highlighting the linguistic diversity within this workforce. All the findings are summarized in Table 1. Thematic analysis findings are summarized in Table 2. A thematic analysis of 218 free-text comments yielded five major themes, reflecting the complex experiences of expatriates.

**Table 1:** Demographic and social attributes of expatriate healthcare professionals in Qatar.

Demographic characteristic	Count	Percentage
Gender		
Male	91	45.70%
Female	108	54.30%
Nationality		
Egyptian	11	5.50%
India	34	17.10%
Jordan	14	7.00%
Pakistan	21	10.60%
Palestine	7	3.50%
Philippines	46	23.10%
Sudan	12	6.00%
Syria	8	4.00%
United Kingdom	34	17.10%
United States	12	6.00%
Age groups (years)		
24-34	40	20.10%
35-44	77	38.70%
45-54	68	34.20%
55-64	11	5.50%
65 and over	3	1.50%
Duration of stay		
Less than 1 year	3	1.50%
1-3 years	71	35.70%
4-7 years	78	39.20%
8-10 years	20	10.10%
More than 10 years	27	13.60%
Job title		
Professional (nurses and doctors)	167	83.90%
Manager	19	9.50%
Clerical role	13	6.50%
Arabic language proficiency		
None	3	1.50%
Basic	116	58.30%
Conversational	7	3.50%
Fluent	73	36.70%

**Table 2:** Thematic analysis of free-text comments. HR: human resources

Theme	Positive comments	Negative comments
Personal and family concerns	Access to advanced medical technologies, supportive human resource policies, respectful workplace, and diverse patient demographics. Access to diverse international communities, excellent healthcare facilities, safe environment for families, and vibrant expat events.	Difficulties with family visas, expensive schooling, and language barriers.
Work-related matters	Quick and efficient emergency services, proactive environmental policies, accessible public amenities, and strong legal system. Access to advanced medical technologies, supportive HR policies, respectful workplace, and diverse patient demographics.	Hierarchical culture, language barriers, heavy workload, and limited advancement opportunities.
Government and system challenges	Easy access to regional travel, vibrant nightlife, and social scene, availability of international goods, and rich historical sites. Quick and efficient emergency services, proactive environmental policies, accessible public amenities, and strong legal system.	Complexity in obtaining residency and work permits, marked by extensive paperwork and bureaucratic processes. Limited English language accessibility on government websites.
Social and cultural challenges	Supportive language learning programs, availability of bilingual resources, encouraging local community, and cultural immersion. Easy access to regional travel, vibrant social life and social scene, availability of international goods, and rich historical sites.	Conservative dress code, cultural gap, language barriers, and feeling of isolation.
Arabic language challenges	Access to advanced medical technologies, supportive HR policies, respectful workplace, and diverse patient demographics. Supportive language learning programs, availability of bilingual resources, encouraging local community, and cultural immersion.	Challenging language, misunderstandings, and isolation due to language barriers.

Personal and Family Concerns

Participants appreciated the support for family relocation but faced challenges like delays in family visas and high schooling costs, emphasizing the need for comprehensive pre-arrival orientations and strong support systems.

Work-Related Matters

While there was contentment with professional conditions, issues like language barriers in clinical settings were highlighted, suggesting the need for language courses and interprofessional training programs.

Government and System Challenges

Participants noted the efficiency of certain government services but also faced bureaucratic hurdles, pointing to a need for streamlined processes and improved English accessibility on government websites.

Social and Cultural Challenges

Varied experiences in cultural adaptation were reported, with some finding community support helpful while others struggled with conservative cultural norms and isolation.

Arabic Language Challenges

Participants shared both the benefits of learning Arabic and the difficulties faced due to language barriers, underscoring the importance of accessible language courses and translation services.

Recommendations

Based on the themes identified, Table 3 presents recommendations for healthcare organizations in Qatar to improve expatriate experiences. These include developing orientation programs, fostering inclusive work cultures, simplifying administrative processes, and offering cultural and language support to facilitate smoother integration into the local community.

**Table 3:** Recommendations for healthcare-employing organizations to improve the experiences of expatriates in Qatar.

Recommendation Categories	Recommendations
1. Personal and family considerations	Employers should develop thorough orientation programs for expatriate families, help with schooling and housing arrangements, and create community networks to support new expatriates in their transition.
2. Work-related matters	Foster an inclusive work culture, provide language support, ensure clear communication about job roles, and offer career advancement opportunities.
3. Government and system challenges	Simplify residency and work permit processes and improve English accessibility on government websites.
4. Social and cultural challenges	Organize cultural orientation sessions, encourage social events for expatriates to integrate with locals, and offer language classes to ease communication barriers.
5. Arabic language challenges	Provide accessible Arabic language courses and reliable translation services to assist expatriates in their professional and personal lives.

The questionnaire detailed in the table in the appendix captured expatriate healthcare professionals' adjustment experiences in Qatar. It covered demographics, job factors, language skills, and social support crucial for understanding their integration. A five-point Likert scale quantitatively assessed adjustment and social support, while open-ended questions yielded rich qualitative data. This blend of quantitative and qualitative analysis offered a holistic view of the expatriate experience in Qatar’s healthcare sector.

## Discussion

This study provides a comprehensive overview of the experiences of healthcare expatriates in Qatar, highlighting a spectrum of rewarding and challenging aspects. The findings, derived from the thematic analysis of free-text comments and the demographic data, highlight the complex nature of expatriate adjustment (Tables 1, 2, and the table in the appendix).

Personal and family concerns

A significant positive aspect noted by participants was the efficient assistance provided by employers in family relocation, which echoes similar findings in existing literature [17,18]. The support in housing and schooling facilitated smoother transitions for families. However, challenges like delays in family arrivals leading to loneliness and anxiety, emphasize the need for more comprehensive support in family relocation processes [5]. This suggests that employers and recruitment agencies should offer extensive pre-arrival orientations and continuous family integration support.

Work-related matters

Participants expressed satisfaction with their working conditions, salaries, and benefits, reflecting a positive view of their professional lives [19]. However, language barriers emerged as a notable challenge, affecting patient care and workplace communication [2]. The study's recommendations for specialized language training and interprofessional workshops aimed to enhance workplace communication, reflecting the need for more inclusive and effective language support strategies (Table 3).

Social and cultural challenges

The binary opposition of enriching experiences and adaptation complexities is evident in the expatriates' social and cultural integration. While some expatriates successfully integrated and found support within the expatriate community, others struggled with adapting to local norms and establishing connections with locals, leading to feelings of isolation [9,11]. The study highlights the importance of cultural orientation programs to help expatriates acclimate to local customs and understand the host culture's social intricacies.

Government and system-related challenges

The study sheds light on the bureaucratic hurdles faced by expatriates, such as difficulties in navigating governmental procedures and limited English-language resources on official websites [9]. Recommendations include streamlining government procedures and enhancing English-language resources, which would create a more expatriate-friendly administrative environment.

Arabic language challenges

Language proficiency emerges as a pivotal factor in expatriate adjustment [13,14]. The study underscores the benefits and challenges associated with Arabic language proficiency in daily and professional life. Providing accessible Arabic language courses would be a proactive approach to enhancing the expatriate experience, supporting findings from other research (Table 3) [20].

In summary, the results from Tables 1-3 and the table in the appendix reveal that while expatriates in Qatar's healthcare sector experience numerous benefits, they also face diverse challenges. Addressing these challenges requires a multifaceted approach involving improved support from employers, enhanced cultural and language training, and streamlined bureaucratic processes. This comprehensive understanding of expatriate experiences can inform strategies to improve expatriate adjustment and integration in Qatar's dynamic and multicultural healthcare environment.

Limitations and future research directions

This study, while providing valuable insights into the experiences of healthcare expatriates in Qatar, has several limitations that warrant mention. The response rate of 51.69%, resulting in 199 participants, may limit the generalizability of the findings. The recruitment method, which relied on WhatsApp groups, could have introduced a selection bias, potentially skewing the sample towards certain demographics and not fully representing the expatriate community. The English-only format of the survey may have excluded non-English-speaking expatriates, potentially overlooking their unique challenges.

As a cross-sectional study, it captures a static picture of experiences without tracking changes over time. The reliance on self-reported data raises concerns about biases such as inaccurate recall and the desire to present oneself favorably. While the thematic analysis based on survey responses (table in the appendix) and free-text comments provided valuable insights, it could not achieve the depth that qualitative methods like in-depth interviews or focus groups might offer (Tables 2, 3). Additionally, the study's focus on the healthcare sector in Qatar may not be fully applicable to other sectors or cultural contexts.

Future research should aim for a wider dissemination of the survey, encompassing a more varied expatriate demographic to enhance representativeness. Longitudinal studies would be beneficial to understand the evolution of expatriate adaptation over time. Incorporating qualitative methodologies, such as in-depth interviews, focus groups, ethnographic studies, and detailed case analyses, could provide a richer, more personal view of the expatriate experience. Expanding the scope to include various professional sectors and linguistic groups would add inclusivity to the research. Employing larger sample sizes and refined psychometric tools could further elucidate the underlying dynamics and long-term implications of expatriate life.

Given these limitations, the findings of this study should be interpreted with a focus on transferability rather than wide-ranging generalizability. Acknowledging the potential biases inherent in the research approach, especially in relation to the unique challenges faced by expatriates in Qatar’s healthcare sector and their cultural and professional integration, is crucial. This recognition paves the way for more nuanced, comprehensive future research in this field.

## Conclusions

This study provides an in-depth analysis of healthcare expatriates' experiences in Qatar, focusing on their adjustment challenges and successes. It identifies key areas like language, culture, bureaucracy, and the importance of support from workplaces and family. These findings offer valuable insights for enhancing expatriate healthcare workers' experiences in Qatar. Despite its limitations, the study encourages further research on expatriate life, emphasizing inclusive and comprehensive methods. It underscores the critical role of support systems in promoting effective expatriate integration and improving healthcare sector outcomes.

## Appendices

**Table 4:** Expatriate adjustment questionnaire.

Demographics and language proficiency:
Gender: male | female | prefer not to say
Age (years): 24-34, 35-44, 45-54, 55-64, and 65 and over (dropdown)
Nationality: Egypt, India, Jordan, Pakistan, Palestine, Philippines, Sudan, Syria, UK, USA (dropdown)
Occupational level: (senior executive to clerical) (dropdown)
Arabic language ability: none | basic | conversational | fluent (dropdown)
Experience in the company: respond on a five-point rating scale from “no one” to “many” would do this
1. Do you have a companion for necessary activities?
2. Is there someone to check on your well-being?
3. Do you have a source for orientation information?
4. Is there assistance with local regulations?
5. Can someone clarify complex situations for you?
6. Does someone inform you of local prohibitions?
7. Is there guidance for unfamiliar tasks?
8. Do you receive help in understanding ambiguities?
9. Is there support for communication challenges?
10. Does someone help you grasp the local culture and language?
11. Are you informed about your options?
12. Do you have company for outings without obligation?
Adjustment in Qatar: rate on a five-point rating scale on your adjustment from “extremely adjusted” to “extremely unadjusted” across various aspects like living conditions, housing, and socializing
1. Overall living conditions
2. Quality of housing
3. Access to food and shopping
4. Affordability of living
5. Availability of entertainment and leisure
6. Quality of healthcare services
7. Clarity of job duties
8. Clear performance metrics
9. Management roles clarity
10. Social life with locals
11. Daily interactions with locals
11. Socializing with locals outside work
12. Ease of communication with locals
Open-ended questions:
Q8: Describe challenges faced as an expat
Q9: Suggestions for new expats
